# Editorial: Immunosenescence and metabolic reprogramming in aging: mechanistic insights and interventions

**DOI:** 10.3389/fcell.2026.1848508

**Published:** 2026-04-28

**Authors:** Tong Zhu, Hong Jiang, Zhaokai Zhou, Ran Xu, Xudong Zhu, Wenxin Li

**Affiliations:** 1 Department of Breast Surgery, Panjin Central Hospital, Panjin, Liaoning, China; 2 Department of Hepatopancreatobiliary Surgery, Cancer Hospital of Dalian University of Technology, Liaoning Cancer Hospital & Institute, Shenyang, Liaoning, China; 3 Department of Urology, The Second Xiangya Hospital of Central South University, Changsha, Hunan, China; 4 National Clinical Research Center for Metabolic Diseases, The Second Xiangya Hospital of Central South University, Changsha, Hunan, China; 5 Markey Cancer Center, University of Kentucky, Lexington, KY, United States

**Keywords:** aging, immunosenescence, interventions, mechanistic insights, metabolic reprogramming

## Immunosenescence and metabolic reprogramming in aging

Aging is a complex biological process characterized by progressive remodeling across multiple physiological systems. Its most prominent features are alterations in immune function and metabolic regulation. Immunosenescence is not regarded solely as a decline in immune defense; rather, it is increasingly recognized as a systemic process marked by chronic, low-grade inflammation, shifts in immune cell composition and function, and reduced tissue repair capacity (Lopez-Otin et al., 2023; Nikolich-Zugich, 2018; Franceschi et al., 2018). Metabolic reprogramming has emerged as a fundamental regulatory mechanism underpinning cellular stress responses, tissue homeostasis, and disease progression (O’Neill et al., 2016; Wiley and Campisi, 2021). In this context, the interplay between immunosenescence and metabolic reprogramming represents a critical entry point for elucidating the mechanisms underlying age-related diseases.

**FIGURE 1:**
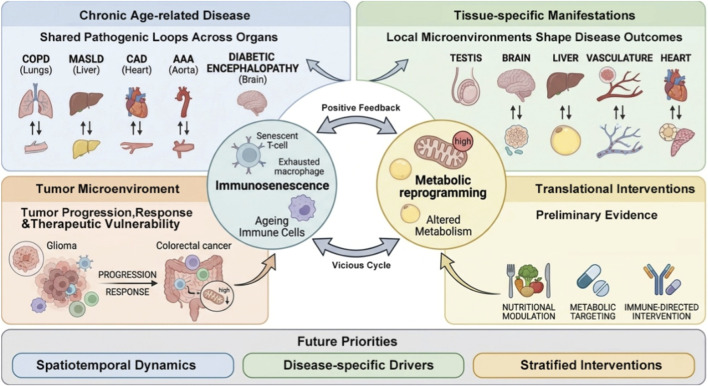
Immunosenescence and metabolic reprogramming as interconnected immunometabolic networks in aging. Aging-associated remodeling promotes both immunosenescence and metabolic reprogramming, which reciprocally interact to drive immunometabolic imbalance. This Research Topic highlights four related dimensions of this process: (i) positive-feedback relationships between immunosenescence and metabolic reprogramming in chronic diseases, (ii) tissue-specific manifestations of this interaction, (iii) its extension into the tumor microenvironment, and (iv) its emerging translational potential. Collectively, these studies support a broader framework for understanding how immunometabolic remodeling contributes to aging-related pathology and provides opportunities for intervention.

Despite growing interest in immune aging and immunometabolism, several key questions remain unresolved. How do immunosenescence and metabolic reprogramming interact across different tissues? Are these interactions conserved across diverse diseases, or are they shaped by specific microenvironmental contexts? To what extent can this immunometabolic imbalance be translated into actionable therapeutic strategies? Addressing these questions, this Research Topic emphasizes the role of the immunometabolic imbalance network in aging and synthesizes evidence from studies on chronic diseases, organ-specific aging, tumor biology, and translational interventions. The discussion is organized into four interrelated perspectives: (i) positive-feedback relationships between immunosenescence and metabolic reprogramming in chronic diseases, (ii) tissue-specific manifestations of this interaction, (iii) its extension into the tumor microenvironment, and (iv) its emerging translational potential in clinical practice (Figure 1).

## Advances in immunosenescence-associated metabolic reprogramming

First, immunosenescence and metabolic reprogramming are not independent phenomena accompanying age-related diseases; rather, they form a mutually reinforcing pathogenic network. This concept is most clearly illustrated in the studies by Chen et al. and Xu et al. Chen et al. proposed an “immune-metabolic positive feedback model” in chronic obstructive pulmonary disease (COPD), emphasizing that chronic inflammation, immune dysfunction, and metabolic abnormalities do not occur in a simple linear sequence but instead interact through reciprocal amplification. Xu et al. elaborated on this framework in metabolic dysfunction-associated steatotic liver disease (MASLD), introducing an age-dependent “immunometabolic vicious cycle” in which immunosenescence, chronic inflammation, and metabolic dysregulation collectively drive disease initiation, progression, and pathological deterioration. Together, these two studies demonstrate that aging-related pathology should not be understood merely as the accumulation of isolated defects but rather as the outcome of self-reinforcing loops linking immune remodeling and metabolic stress. This framework was further extended by research on coronary artery disease, abdominal aortic aneurysm, and diabetic encephalopathy. In coronary artery disease, Bie and Tao showed that immune aging contributes to a chronic cardiovascular injury environment. From the perspective of metabolic reprogramming in abdominal aortic aneurysm, Wu et al. highlighted the close interconnection between metabolic abnormalities and local inflammatory and vascular microenvironments. Huai et al. extended this logic to the nervous system, emphasizing that metabolic reprogramming may be a key driver of accelerated brain aging and cognitive decline in diabetic encephalopathy. Collectively, these studies indicate that the positive-feedback relationship between immunosenescence and metabolic reprogramming is not restricted to a single disease but may represent a common pathological basis across pulmonary, hepatic, cardiovascular, vascular, and neurological disorders.

Second, although immunosenescence and metabolic reprogramming share common mechanisms across diseases, their manifestations are strongly shaped by tissue-specific microenvironments. This aspect is particularly illustrated by Zhan et al. ‘s study on testicular immunosenescence. By focusing on the reproductive system, Zhan et al. demonstrated that immunosenescence affects not only classical immune functions but also contributes directly to age-related spermatogenic decline, suggesting that local immune dysregulation in the testis may represent a key mechanism of male reproductive aging. This contribution broadens the scope of the Research Topic by showing that immunometabolic imbalance is not confined to highly inflammatory chronic diseases but can also influence specialized tissue niches with distinct structural and functional demands. The tissue-specific nature of this process is further supported by the studies of Huai et al., Wu et al., and Bie and Tao. In diabetic encephalopathy, metabolic reprogramming is linked to accelerated brain aging and cognitive decline, underscoring the sensitivity of the nervous system to immunometabolic imbalance. In abdominal aortic aneurysm, metabolic abnormalities interact with the remodeling of the vascular microenvironment, thereby promoting the progression of vascular wall lesions. In coronary artery disease, immunosenescence appears to play a disease-specific pathophysiological role, reflecting the close association between immune aging and chronic injury in the cardiovascular system. Collectively, these studies suggest that while immunosenescence and metabolic reprogramming may represent a shared framework of aging, their biological outcomes are ultimately shaped by the local ecology of different tissues.

Third, this Research Topic extends the significance of immunometabolic imbalance into the tumor setting. Using glioma as an example, Fan et al. emphasized that metabolic reprogramming and immunosenescence should not be regarded as separate lines of investigation but as interconnected factors jointly shaping the tumor microenvironment in therapeutic contexts. This perspective suggests that understanding the coupling between these processes may open new avenues for glioma therapy. In parallel, Zhu et al. expanded this logic to colorectal cancer, highlighting the dual significance of metabolic remodeling and immune aging in both tumor biology and therapeutic implications. Their findings indicate that the interaction between these processes influences not only tumor progression but also treatment response and clinical intervention strategies. Taken together, these studies suggest that in cancer, the relationships between immunosenescence and metabolic reprogramming are not merely general features of aging but key determinants of tumor microenvironmental status and therapeutic vulnerability. In this way, the Research Topic naturally extends from age-related tissue injury to tumor progression and precision treatment.

Finally, the studies collected here point toward emerging opportunities for intervention. Pritchard et al.‘s original research moves the discussion beyond mechanistic interpretation toward practical application by examining inflammatory and mitochondria-related pathways in adipose tissue from older overweight adults Pritchard et al. This work suggests that nutritional supplementation may hold exploratory value in modulating immunometabolic imbalance. At the same time, several review articles in this Research Topic emphasize potential intervention strategies, therapeutic opportunities, and clinical implications within their respective disease contexts. These contributions indicate that the Research Topic does not stop at describing associations between immunosenescence and metabolic reprogramming but is increasingly directing attention toward intervention targets, therapeutic strategies, and clinical translation. Nevertheless, current evidence remains largely preliminary. A critical future challenge will be to develop interventions that are more precise, stable, and reproducible, particularly in the context of tissue complexity and disease heterogeneity.

## Conclusion and perspectives

In summary, the studies collected in this Research Topic suggest that immunosenescence and metabolic reprogramming should not be viewed as two parallel lines of age-related change, but rather as components of an interacting, dynamically remodeled, and potentially targetable immunometabolic network. This framework provides a strong explanatory value across chronic inflammatory diseases, organ-specific aging, tumor biology, and translational research. Future investigations should prioritize at least three key issues: (i) the spatiotemporal dynamics of this network within different cell types and tissue microenvironments, (ii) the principal drivers of immunometabolic imbalance in distinct disease settings, and (iii) the development of more precise stratified intervention strategies informed by these mechanisms. Addressing these questions will enable research on immunosenescence and metabolic reprogramming to progress from conceptual integration to actionable mechanistic translation.
